# Physico-chemical characterization and pharmacological evaluation of sulfated polysaccharides from three species of Mediterranean brown algae of the genus *Cystoseira*

**DOI:** 10.1186/s40199-015-0089-6

**Published:** 2015-01-13

**Authors:** Hiba Hadj Ammar, Sirine Lajili, Rafik Ben Said, Didier Le Cerf, Abderrahman Bouraoui, Hatem Majdoub

**Affiliations:** Laboratoire des Interfaces et des Matériaux Avancés (LIMA), Faculté des Sciences de Monastir, Université de Monastir, Bd. de l’environnement, 5019 Monastir, Tunisia; Laboratoire de développement chimique, galénique et pharmacologique des médicaments, Faculté de Pharmacie de Monastir, Université de Monastir, 5000 Monastir, Tunisia; Institut National des Sciences et Techniques de la Mer (INSTM), Salambôo, Tunis Tunisia; Université de la Normandie, Laboratoire Polymères Biopolymères Surfaces, UMR 6270 CNRS Université de Rouen, FRE 3101 CNRS, 76821 Mont Saint Aignan, France

**Keywords:** Fucoidans, *Cystoseiraceae*, *Cystoseira*, SEC/MALS/VS/DRI, Anti-inflammatory activity, Gastroprotective activity

## Abstract

**Background:**

Seaweed polysaccharides are highly active natural substances having valuable applications. The present study was conducted to characterize the physico-chemical properties of sulphated polysaccharides from three Mediterranean brown seaweeds (*Cystoseira sedoides*, *Cystoseira compressa* and *Cystoseira crinita*) and to evaluate their anti-radical, anti-inflammatory and gastroprotective activities.

**Methods:**

The different rates of neutral sugars, uronic acids, L-fucose and sulphate content were determined by colorimetric techniques. The different macromolecular characteristics of isolated fucoidans were identified by size exclusion chromatography equipped with a triple detection: multiangle light scattering, viscometer and differential refractive index detectors, (SEC/MALS/VD/DRI). Anti-inflammatory activity was evaluated, using the carrageenan-induced rat paw edema test in comparison to the references drugs Acetylsalicylate of Lysine and Diclofenac. The gastroprotective activity was determined using HCl/EtOH induced gastric ulcers in rats and to examine the antioxidant effect of fucoidans in the three species, the free radical scavenging activity was determined using 1,1-diphenyl-2-picrylhydrazyl.

**Results:**

The pharmacological evaluation of the isolated fucoidans for their anti-inflammatory, and their gastroprotective effect established that these products from *C. sedoides*, *C. compressa* and *C. crinita* exhibited a significant anti-inflammatory activity at a dose of 50 mg/kg, i.p; the percentages of inhibition of the oedema were 51%, 57% and 58% respectively. And, at the same dose, these fucoidans from *C. sedoides* and *C. compressa* showed a significant decrease of the intensity of gastric mucosal damages compared to a control group by 68%, whereas, the fucoidan from *C. crinita* produced a less gastroprotective effect. Furthermore, the isolated fucoidans exhibited a radical scavenging activity.

**Conclusion:**

The comparative study of fucoidans isolated from three species of the genus *Cystoseira* showed that they have similar chemicals properties and relatives anti-radical, anti-inflammatory and gastroprotective activities which are found to be promising.

## Background

Brown seaweeds represent a rich sources of several nutraceuticals components like laminarans, fucoidans, and polyphenols. Among these, fucoidans, a sulphated polysaccharide have been the subject of much interest in recent years, mainly due to their pharmacological and biological potential with anti-viral [1], anti-cancer [2], liver protection [3], anti-inflammatory [4] and antibacterial [5] properties and it also can affect the secretion of extracellular matrix proteins [6] and activate apoptosis [7]. Several studies have attempted to determine the exact structure of fucoidans but only a few examples of regularity in the structure were found. Links, ramifications, the position of the sulphates and other sugars appear to be variables [8]. Fucoidans are generally linear, mainly composed of repeated units of fucoses sulfated at C-2 and/or C-4 with a-(1–3) and/or a-(1–4) linkages [9]. They can also contain uronic acid, optionally acetylated and other neutral sugars such as D-galactose, D-xylose, D-glucose, D-mannose. However, this chemical composition varies depending on the algal specie and it can vary even within the same species. In this paper, we will focus on a comparative study of physico-chemical and biological properties of fucoidans from three species of brown algae of the genus *Cystoseira: C. sedoides, C. compressa* and *C. crinita*. Our attention is particularly paid to this genus of algae for its abundance in the Mediterranean area and more specifically on the Tunisian coast sides. Furthermore, the only structural features of sulphated fucans from this genus of brown seaweed *Cystoseira indica* have been reported by (Mandal et al.) [10].

## Methods

### Sample collection

Brown seaweeds, (*C. crinita, C. compressa, and C. sedoides*) were harvested from the Mediterranean sea, from various areas of the coastal region of Monastir and Tabarka (Tunisia), in June 2007, at a depth between 1 and 3 m. These brown algae are of the family of *Cystoseiraceae*. After collection, the seaweeds were rinsed with fresh water to remove associated debris and epiphytes. The cleaned material was then air dried in the shade at 30°C. The dried samples were finally powdered and stored at – 20°C until use. Identification of specimens was carried out in the National Institute of Marine Sciences and Technologies (Salambôo, Tunisia).

### Extraction of crude polysaccharides

The milled sample was soaked in Methanol-Dichloromethane (1:1) at room temperature for 48 h then filtered. This process was repeated three times. A sequential extraction of seaweed’s powders was carried out with petroleum ether then acetone in a soxhlet apparatus to remove lipophilic pigments (such as chlorophylls) and low molecular weight proteins. Depigmented dried seaweeds were treated three times with 2% aqueous solution of CaCl_2_ during 3 hours, in order to precipitate alginates. After centrifugation, the supernatant enclosing the fucoidans was recovered and then purified by dialysis through tubing of molecular weight cut off 30 KDa and then lyophilized.

### Chemical composition

Total carbohydrates were determined for all the extracted polysaccharides by the phenol – H_2_SO_4_ method [11] using galactose as a standard. Whereas, uronic acids were determined using carbazole method [12] and glucuronic acid as a standard. The sulphate content of the polysaccharides was determined by the turbidimetric method using sodium sulphate (Na_2_SO_4_) as a standard after hydrolyzing the polysaccharides in 2 M HCl at 100°C for 2 h [13]. The content of L-fucose units in fucoidans was estimated by a colorimetric assay with L-cysteine [14]. FTIR were performed in KBr pellets (1 mg polysaccharide in 100 mg KBr). The spectra were recorded on a Perkin Elmer 1600 FTIR spectrometer from 400 to 4000 cm^−1^.

### Molecular weight determination

Analysis of various samples was performed using size exclusion chromatography (SEC) equipped with a triple detection: multi-angle light scattering (MALS) (Down HELEOS II, Wyatt Technology, Ca, USA), viscometer detector (VD) (Viscostar II, Wyatt Technology, Ca, USA) and differential refractive index (DRI) (RID 10 A Shimadzu, Japan). The SEC system consists of a pump (LC10 Ai Shimadzu, Japan) at a flow rate 0.5 mL/min and two columns OHPAK SB 804 and 806 HQ.

The samples were dissolved in the eluent (LiNO_3_ 0.1 mol/L) at 2 g/L. The dissolution was carried out by stirring at 380 rpm for 24 h at room temperature. 3 mL solutions were filtered through membrane 0.45 microns (regenerated cellulose) before injection.

The analyzes were performed by a data processing Zimm [15] “order 1” using angles from (from 34.8° to 142.8°). The corresponding value of dn/dc, in our case is about 0.15 mL/g, the typical value for a polysaccharide [16]. The Astra 6.0.1.7 software package is set to collect and extrapolate data with the aim to obtain for each elution volume the molecular weight and the gyration radius. With an integration of the peak, we calculated the number (Mn) and weight (Mw) average molecular weight and the z-average gyration radius.

The differential viscosimeter detector permits to obtain for each elution fraction the intrinsic viscosity. An integration of the peak gives the average intrinsic viscosity, which allowed us to obtain the average hydrodynamic volume (Vh) using the Einstein − Simha equation:

$$ \mathrm{V}\mathrm{h}=\left[\eta \right]\mathrm{M}/\nu {\mathrm{N}}_{\mathrm{A}} $$

where N_A_ is Avogadro’s number, M is the molar mass, [η] is the intrinsic viscosity (g mL^−1^), and ν is a conformational parameter that takes the value of 2.5 in the case of a spherical conformation.

### DPPH (1,1-diphenyl-2-picrylhydrazyl) radical scavenging activity

To examine the antioxidant effect of fucoidans in the three species, the free radical scavenging activity was determined using DPPH according to the method of Kim et al. [17]. A dilution series of the extracted samples was prepared (0, 0.25, 0.5, 0.75 and 1 mg/mL). A 1 mL volume of each sample was mixed with 1 ml of 30 mmol/L DPPH-ethanol solution. The reaction mixture was then stirred vigorously for 10 seconds using the vortex. Color was allowed to develop in the dark for 30 min. The absorbance is measured at 517 nm against the blank. Radical scavenging activity is expressed as the inhibition percentage and was calculated using the following formula:

$$ \mathrm{Radical}\ \mathrm{scavenging}\ \mathrm{capacity}\left(\mathrm{R}\mathrm{S}\mathrm{C},\ \%\right)=1-\left[\left({\mathrm{A}}_{\mathrm{sample}}\hbox{--} {\mathrm{A}}_{\mathrm{sample}\ \mathrm{blank}}\right)/{\mathrm{A}}_{\mathrm{control}}\right]\times 100. $$

Where the A _control_ is the absorbance of the control (DPPH solution without sample), the A_sample_ is the absorbance of the test sample (DPPH solution plus test sample), and the A_sample blank_ is the absorbance of the sample only (sample without DPPH solution).

### Pharmacological evaluation

#### Animals

All experiments were performed according to the guidelines established by the European Union on Animal Care (CCE Council 86/609). Wistar rats (150 – 200 g) of both sexes purchased from Pasteur Institute (Tunis, Tunisia) were used. They were housed in groups of eight to ten animals in plastic cages at 20-25°C and maintained on a standard pellet diet with free access to water. Animals were fasted for 24 h before the experiments.

### Anti-inflammatory activity

The anti-inflammatory activity of isolated fucoidans was evaluated using the carrageenan induced rat paw oedema test. Wistar rats were divided into groups of six animals and the oedema was induced by injecting 0.05 ml of 1% carrageenan subcutaneously into the sub-plantar region of the left hind paw [18]. Isolated fucoidans from *C. crinita, C. sedoides* and *C. compressa* (25 or 50 mg/kg) and reference drugs were administered intraperitoneally (i.p.) 30 min before the injection of carrageenan. The control group received the vehicle (Saline water 2.5 ml/kg, i.p.). The reference groups received Diclofenac (10 mg/kg, i.p) and (ASL, 300 mg/Kg, i.p.).

Measurement of paw size was done by means of volume displacement technique using Plethysmometer (Ugo Basile no. 7140) immediately before carrageenan injection and 1, 2, 3, 4 and 5 h after carrageenan injection. Percentages of inhibition in our anti-inflammatory tests were obtained for each group using the following ratio:

$$ \left[{\left(\mathrm{V}\mathrm{t}-\mathrm{V}\mathrm{o}\right)}_{\mathrm{control}} - {\left(\mathrm{V}\mathrm{t}-\mathrm{V}\mathrm{o}\right)}_{\mathrm{treated}}\right] \times 100/{\left(\mathrm{V}\mathrm{t}-\mathrm{V}\mathrm{o}\right)}_{\mathrm{control}} $$

Where, Vt is the average volume for each group and Vo is the average volume obtained for each group before any treatment [19].

### Gastroprotective activity

The gastroprotective activity of fucoidans from three species of genus *Cystoseira* was studied in HCl/EtOH induced gastric ulcer [20]. Rats were divided into different groups, fasted for 24 h prior receiving an intraperitoneal injection of the isolated fucoidans (25 or 50 mg/kg). Two other groups received Ranitidine (60 mg/kg, i.p.) and Omeprazole (30 mg/kg, i.p.) as reference drugs. After 30 min, all groups were orally treated with 1 ml/100 g of 150 mM HCl/EtOH (40:60, v/v) solution for gastric ulcer induction. Animals were sacrificed 1 h after the administration of ulcerogenic agent; their stomach were excised and opened along the great curvature, washed and stretched on cork plates. The surface was examined to detect the presence of lesions and to measure their extent. The summative length of the lesions along the stomach was recorded (mm) as lesion index.

### Statistical analysis

Results were analyzed using One Way ANOVA (Fisher LSD post hoc test) and expressed as mean ± s.e.m, using SPSS Statistics Software (SPSS for Windows software release 18.0). Difference between means of treated and control groups were considered significant at P < 0.05.

## Results and discussion

### Extraction and chemical analysis

The main concern in the isolation procedures of fucoidans was to avoid their contamination with other polysaccharides, like laminaran and especially alginic acid. The hot extraction, in the presence of CaCl_2_ was allowed to separate the insoluble calcium alginate from the soluble fraction. This fraction is rich in fucoidan and laminaran. To eliminate this latter we had to recourse to dialysis.

The extraction yields show almost no difference between seaweed species (about 3%) (Table 1). The yields obtained were in good agreement with Rioux et al. [9] for other seaweed species (*Ascophyllum nodosum* (3.3%)*, Fucus versiculosus* (4%)*, Saccharina longicruris* (2.6%)).

**Table 1:** **Yields of extraction and carbohydrates analysis**

		Yield* (%)	**Total sugar (%)**	**Uronic acid (%)**	**Sulfate (% SO**_**3**_ **Na)**	**Fucose (%)**
Fucoidans	*C. sedoides*	3.3	21.3	5.9	16.3	54.5
	*C. compressa*	3.7	13.0	9.3	16.6	61.5
	*C. crinita*	2.8	44.5	13.8	15.7	43.4

*Yields of extraction given in % of dry weight.

The different colorimetric assays confirm that isolated polysaccharides are fucoidans, mainly composed by fucose (43 to 61%). The amount of sulphates was determinated for the isolated fucoidans. There was no difference found between the three species; an average of 16% was observed for all. Besides, the isolated fucoidans were moderately sulphated compared to those of *Cystoseira indica* (11.5%) [10]*, Saccharina longicruris* (12%) [9], *Fucus vesiculosus* (12%) [21] and *Sargassum stenophyllum* (28%) [22].

The FTIR spectrums of the isolated polysaccharides show typical absorption bands of fucoidan. Their exact absorption peaks are given in (Table 2). The intensity of the bands at 3400–3200 cm^−1^ was assigned to the deformation of O-H. The bands between 3000 and 2925 cm^−1^ were attributed to the C-H stretching frequency and the strong absorption at approximately 1050 cm^−1^ corresponded to the C-O-C stretching frequency of the glycosidic bonds [23]. Besides, the extracted fractions showed all absorption at 1650–1620 cm^−1^, indicating the presence of uronic acid. The characteristic absorption bands of fucoidan are those who indicate the presence of sulphate (SO_4_) and methyl (CH_3_) groups, as fucoidan contains mainly fucose [24], which is a monosaccharide that has a methyl group attached to the C5 position. The signals at 1457–1423 cm^−1^ were attributed to the asymmetrical bending vibration of CH_3_. The strong absorption band at 1255–1240 cm^−1^ (S = O stretching) confirms the presence of a significant amount of sulfate in the polysaccharides. The sharp band at 820 cm^−1^ (C-S-O) suggest that the majority of sulphate groups occupy positions 2 and/or 3 (equatorial positions) [25].

**Table 2:** **The most diagnostic peaks in the IR spectra of extracted polysaccharides**

**Fucoidans**			**Assignment**
***C. compressa***	***C. crinita***	***C. sedoides***	
3421 cm^−1^	3411 cm^−1^	3412 cm^−1^	O-H assoc. stretching vibration
2925 cm^−1^	2925 cm^−1^	2928 cm^−1^	C-H stretching vibration
1652 cm^−1^	1641 cm^−1^	1639 cm^−1^	C = O stretching vibration
1457 cm^−1^	1441 cm^−1^	1423 cm^−1^	asymmetrical bending vibration of CH_3_
1263 cm^−1^	1231 cm^−1^	1231 cm^−1^	S O stretching vibration
1049 cm^−1^	1050 cm^−1^	1050 cm^−1^	C-O-C stretching vibration
826 cm^−1^	825 cm^−1^	825 cm^−1^	C–O–S vibration

**Figure 1:**
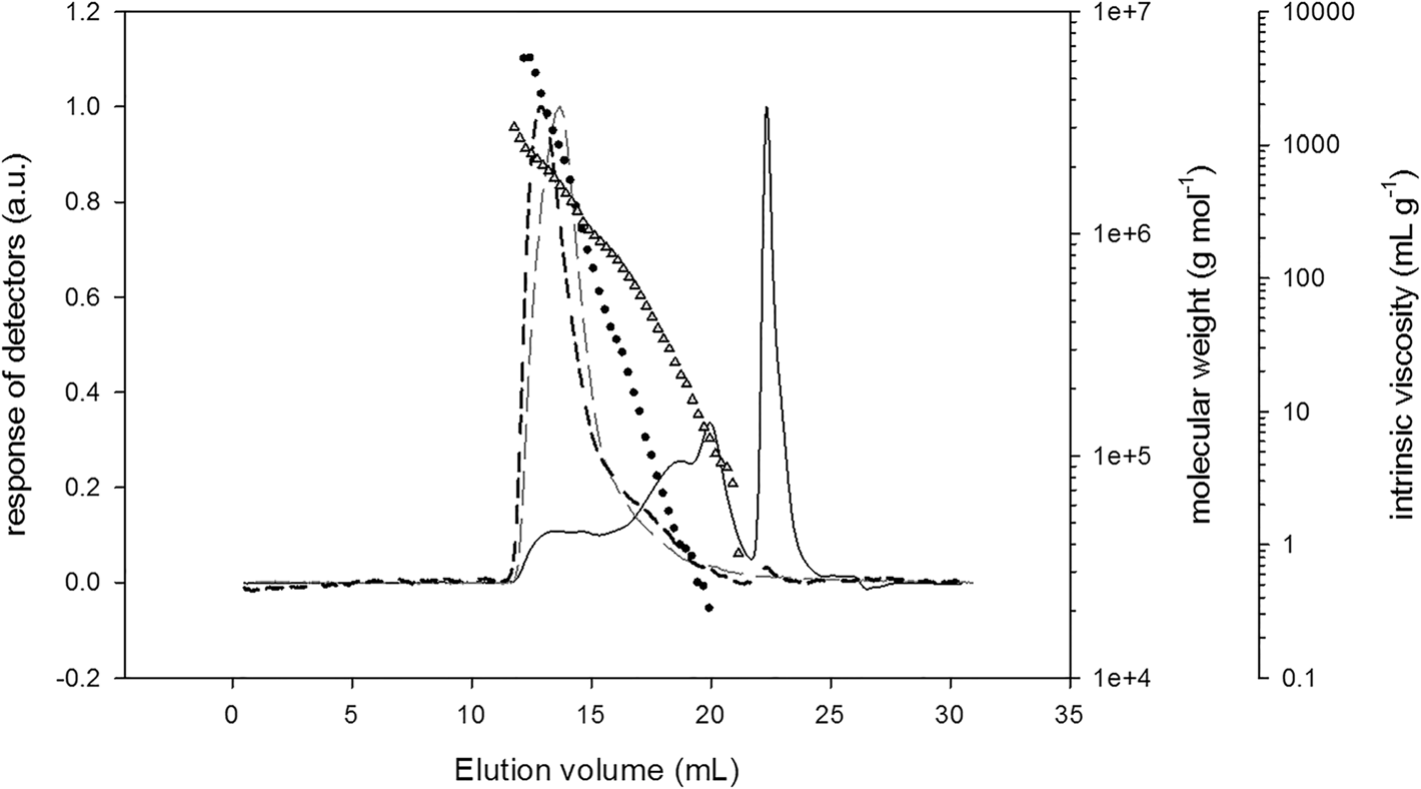
**Elution profiles of** ***C. sedoides*** **sample with Mw and [η] distribution determined by SEC/MALS/VS/DRI in 0.1 mol L** ^**−1**^ **LiNO**_**3**_ **aqueous solution.** Differential refractive index (full line), light scattering at 90° (dotted black line), specific viscosity (dotted grey line), Mw: molecular weight (black circles) and [η] intrinsic viscosity (grey triangle).

SEC/MALS/VD/DRI experiments were carried out in 0.1 mol/L LiNO_3_ to determine molecular weights and size information of biopolymers studied. As an example, we have reported on (Figure 1) the elution profiles and the molecular weight and intrinsic viscosity distribution of *C. sedoides* sample. The Polysaccharides are eluted between 11 and 22 mL showing a large distribution. After 22 mL the peak obtained with refractive index detector is due to donnan effect on salt. Firstly, between 11 and 16 mL, light scattering and viscometric responses are intensive with a relative low concentration response (DRI). Consequently, *C. sedoides* have very long macromolecular chains with molecular weight up the 1 000 000 g.mol^−1^. An other population is eluted from 16 mL to 22 mL. The DRI exhibits high intensity with low intensity for MALS and DV detectors. The molecular weights are lower and it is no possible to obtain gyration radii due to isotropic diffusion. Nevertheless, we can estimate hydrodynamic radii all along the elution volume with viscometric data. In conclusion, SEC/MALS/VD/DRI analysis permits to obtain some characteristics of whole sample as Mn, Mw, polydispersity index (Đ) weight average (Rh) and [η].

**Figure 2:**
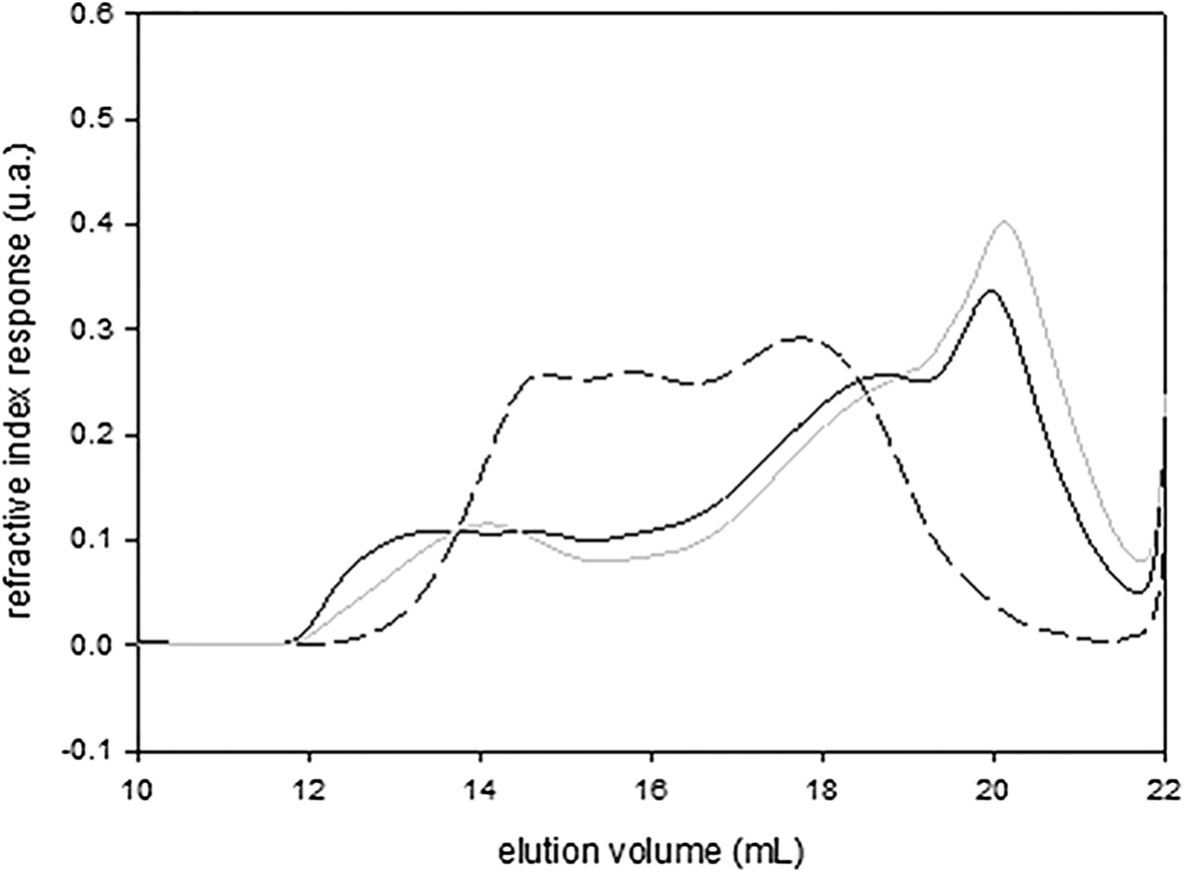
**Elution profiles from differential refractive index of different extract determined by SEC/MALS/VS/DRI in 0.1 mol L** ^**−1**^ **LiNO**_**3**_ **aqueous solution.** C. sedoides (full black line), C. compressa (dotted black line) and C. crinita (full grey line).

Same analysis was made on the two other fucoidan samples. We have reported only the refractive index response for a better view of the three samples (Figure 2). *C. sedoides* and *C. crinita* are separated in the same way. They have similar molecular repartition. *C. compressa* has a shorter distribution of molecular weight with a peak between 13 and 20 mL. All results are summarized in (Table 3). The presence of very short chain in the two samples *C. sedoides* and *C. crinita* decreases drastically the Mn and consequently increases the polydispersity. In the same way, very high molecular weights obtained at the beginning of the peak (between 12 and 13 mL) influence greatly the intrinsic viscosity and average hydrodynamic diameter, which are obtained by weight average.

**Table 3:** **Average macromolecular characteristics of fucoidans isolated from** ***C. crinita, C. compressa,*** **and** ***C. sedoides*** **) determined by SEC/MALS/VD/DRI (0.1 mol/L LiNO**_**3**_ **)**

		**Mn (g/mol)**	**Mw (g/mol)**	Đ*	**[η] (mL.g** ^**−1**^ **)**	**R**_**H**_ **(nm)**	a**
Fucoidans	*C. sedoides*	26 000	642 000	24	133	18	0.73
	*C. compressa*	114000	545 000	4.8	92	14	0.70
	*C. crinita*	18000	339 000	18.2	125	18	0.84

*Đ = Mw/Mn, polydispersity index.

**a: the Mark Houwink exponent.

The knowledge of molecular weight and intrinsic viscosity for each elution volume can be used to determine the Mark-Houwink exponent. Values between 0.7-0.8 are in agreement with random coil conformation [26].

However, it’s important to note that chemical composition, molecular weight and structure varies depending on the source of fucoidans, the harvest period and the extraction methods.

### DPPH radical scavenging activity

DPPH is a stable radical that can directly react with anti-oxidants. It has been used extensively as a free radical to evaluate reducing substances and is a useful reagent for investigating the free radical scavenging. When the DPPH radical is scavenged by anti-oxidants through the donation of hydrogen to form a stable DPPH-H molecule, the color changes from purple to yellow. In this work, DPPH free-radical scavenging effect of each sample was calculated and the EC_50_ values were presented in (Table 4). Fucoidans from different species (*C. crinita, C. compressa and C. sedoides*) exhibited DPPH radical scavenging activity with an EC50 value of 0.76 mg/mL, 0.84 mg/mL and 0.96 mg/mL, respectively. These scavenging effects of fucoidans, were less important than produced by the reference compound, Ascorbic acid and decreased in this order *C. crinita > C. compressa > C. sedoides.*

**Table 4:** **EC50 values of fucoidans extracted from** ***C. crinita, C. compressa, and C. sedoides*** **in radical scavenging activity**

	**Ascorbic acid**	***C. sedoides***	***C. compressa***	***C. crinita***
EC50*(mg/ml)	0.13 ± 0.01	0.96 ± 0.01	0.84 ± 0.06	0.76 ± 0.04

*EC50 value: the effective concentration at which the antioxidant activity was 50%; the absorbance was 0.5 for reducing power; 1,1-diphenyl-2-picrylhydrazyl (DPPH).

### Anti-inflammatory activity

Carrageenan has been widely used as a noxious agent to induce experimental inflammation for the screening of compounds possessing anti-inflammatory activity. This phlogistic agent, when injected locally into the rat paw, induced a severe inflammatory reaction, discernible within 30 min [27]. Administration of fucoidans of *C. sedoides, C. crinita* and *C. compressa* (25 and 50 mg/kg, i.p.) produced a significant reduction of oedema throughout the period of observation in a dose-related manner. The experimental results are shown in (Table 5). All the isolated fucoidans showed significant anti-inflammatory activity; in fact treatment with sulphated polysaccharides from *C. sedoides, C. compressa* and *C. crinita* (at the dose of 50 mg/kg, i.p.) inhibited the formation of the oedema by 51%, 56.81% and 58.21%, respectively, 3 h after the administration of carrageenan. Results were statistically significant compared to the control and are quite similar to those observed for both the group treated with Diclofenac (10 mg/kg) and ASL (acetylsalicylic of lysine 300 mg/kg) which inhibited oedema formation by 55.07% and 56.81%, respectively. Carrageenan induced inflammation in a biphasic phenomenon [28]. The first phase of oedema is attributed to release of histamine and 5-hydroxytryptamine and the second accelerating phase of swelling is attributed to prostaglandin like substances. The knowledge of these mediators involved in different phases is important for interpreting the mode of fucoidan action. Fucoidans might have inhibited the release or actions of the various chemical mediators such as histamine, 5-HT, kinins, and prostanoids known to mediate acute inflammation induced by phlogistic agents.

**Table 5:** **Effect of the administration of fucoïdanes isolated from** ***C. sedoides, C. compressa*** **and** ***C. crinita*** **and reference drugs in carrageenan induced rat paw edema**

**Samples**		**Dose (mg/kg)**	**Edema (mL × 10** ^**−2**^ **)**			Edema inhibition (%)*		
			**1 h**	**3 h**	**5 h**	**1 h**	**3 h**	**5 h**
Control		-	26 ± 2.75	57.5 ± 1.51	59 ± 3.27	-	-	-
ASL (reference)		300	17.66 ± 1.63	24.83 ± 1.72	29.33 ± 1.21	32.07	56.81	50.28
Diclofenac (reference)		10	15.66 ± 5.12	25.83 ± 2.40	28.66 ± 3.61	39.76	55.07	51.42
Fucoidans	*C. sedoides*	25	13.66 ± 3.20***	29.66 ± 5.20***	40.16 ± 4.70***	47.46^a^	48.41^a^	31.93^a^
		50	10.66 ± 4.08***	28.16 ± 3.38***	41.66 ± 1.21***	59.00^b^	51.02^a^	29.38^a^
	*C. compressa*	25	12.66 ± 2.73***	26.83 ± 1.04***	29.83 ± 2.40***	51.30^a^	53.33^a^	49.44^a^
		50	11.83 ± 4.09***	24.83 ± 2.48***	28.00 ± 2.36***	54.50^a^	56.81^a^	52.54^a^
	*C. crinita*	25	12.66 ± 3.43***	26.50 ± 3.39***	19.83 ± 1.94***	51.28^a^	54.17^a^	66.38^a^
		50	13.20 ± 2.68***	24.16 ± 2.56***	28.50 ± 2.50***	49.23^a^	58.21^a^	51.69^b^

Data are expressed as mean ± s.e.m. (n = 6).

^*^Mean values with different superscript letters in the same row are significantly different at p ≤ 0.05.

***p < 0.001.

### Gastroprotective activity

The results of gastroprotective activity of the isolated compounds from *C. compressa, C. sedoides* and *C. crinita* on gastric ulcer induced by HCl/ethanol solution are shown in (Table 6). Oral administration of the damaging agent to the control group clearly produced a mucosal damage characterized by multiple hemorrhage red bands of different sizes along the long axis of the glandular stomach. Pretreatment with fucoidans of *C. compressa*, *C. sedoides* and *C. crinita* (25, 50 mg/kg, i.p.) produced significant decrease in the intensity of gastric mucosal damages induced by the necrotizing agent HCl/EtOH compared with control group. Fucoidans from *C. compressa* and *C. sedoides* at the dose of 50 mg/kg produced an important protective effect against gastric mucosal lesion which is quite similar to the effect produced by the reference drug, ranitidine. The percentage of inhibition of ulcer were 68.18%, 68.51% respectively for fucoidans from *C. compressa* and *C. sedoides.* However, fucoidan from *C. crinita* showed less protection (59.90% of inhibition).

**Table 6:** **Results of antiulcerogenic activity of fucoïdanes isolated from** ***C. sedoides, C. compressa*** **and** ***C. crinita*** **on gastric ulcer induced by HCl/ethanol solution**

**Samples**		**Dose (mg/kg)**	**Average lesion (mm)**	Ulcer inhibition (%)*
Control		_	50.33 ± 5.50	_
Ranitidine (reference)		60	43.38 ± 4.35	66.96
Oméprazole (reference)		30	17.50 ± 1.38	86.67
Fucoidans		25	20.83 ± 2.56***	58.57 ± 5.09^a^
		50	16.00 ± 2.19***	68.19 ± 4.35^b^
	*C. sedoides*	25	22.83 ± 3.70***	54.60 ± 7.37^a^
		50	15.83 ± 4.60***	68.51 ± 5.24^b^
	*C. crinita*	25	28.33 ± 4.09***	43.66 ± 5.13^a^
		50	20.17 ± 6.17***	59.90 ± 7.26^b^

Data are expressed as mean ± s.e.m. (n = 6).

*Mean values with different superscript letters in the same row are significantly different at p ≤ 0.05.

***p < 0.001.

HCl-ethanol induced gastric mucosal lesions may be multifactorial, with static blood flow contributing significantly to the hemorrhagic as well as the necrotic aspects of the tissue injury [29]. The decrease in the number of lesions may be due the reduction in the levels of gastric secretion [30]. The alteration in the acidity/volume of the gastric juice is due to the production of HCl, which may increase the permeability of the mucosal membrane [31]. Thus, the overall protection by fucoidans against HCl-ethanol induced gastric ulceration in experimental rats suggest that it contains some anti-ulcer agents that may hasten the decomposition of free radicals generated, thereby strengthening the gastric mucosal antioxidant defense system suggesting an gastroprotective effect of fucoidans from brown algae.

## Conclusion

The comparative study of fucoidans isolated from three species of the genus *Cystoseira* showed that they have similar properties regarding the percentage in sulphates, L-fucose content and their molecular weight. However this requires an advanced structural study to determine the length of the general chain and the branching of fucoidans.

These similarities are reflected on pharmacological activities; in fact the different isolated fucoidans have similar anti-radical, anti-inflammatory and gastroprotective activities which are found to be promising.

## Abbreviations

ASL: Acetylsalicylic of lysine
C. sedoides: *Cystoseira sedoides*
C. compressa: *Cystoseira compressa*
C. crinita: *Cystoseira crinita*
Mn: Average molecular number
Mw: Average molecular weight
Rh: Average hydrodynamic diameter
[η]: Intrinsic viscosity
Đ: Polydispersity index
